# Trends in Self-Harm, Aggression, and Readmission Risk in Adolescent Inpatient Psychiatric Care: A temporal trends analysis across the COVID-19 period

**DOI:** 10.1192/j.eurpsy.2026.10546

**Published:** 2026-07-31

**Authors:** S. Webb, R. Morris, A. Muller, J. Forsyth, M. Zuccala

**Affiliations:** 1 School of Psychology; 2 Faculty of Medicine and Health, The University of Sydney; 3 Brolga Adolescent Inpatient Unit, Northern Sydney Local Health District, Sydney, Australia

## Abstract

**Introduction:**

Adolescent psychiatric inpatient units faced heightened service challenges during the COVID-19 pandemic, with rising admissions for deliberate self-harm (DSH) and suicide risk. Few studies have examined how systemic and contextual factors influenced risk within these units during the peak of service disruptions, and in the post-pandemic period.

**Objectives:**

This study presents a longitudinal analysis of incidents on an adolescent psychiatric inpatient unit in Sydney, Australia between January 2020 and December 2023. It aims to understand the effect of COVID-19 and associated practice changes on readmission and incident rates on the ward.

**Methods:**

Poisson and binomial regression models tested temporal trends in DSH, aggression and absconding incidents across the study period. Patient-level (gender, age, admission history), ward-level (readmission rate, average length of stay, bed occupancy), and COVID-19 (community and ward lockdown status) variables were included.

**Results:**

Incident rates increased significantly over time, with the steepest rise observed for DSH, particularly among female patients and those with multiple admissions. A dose-response relationship emerged, with DSH incidents rising from 11% at first admission to 87% at nine or more readmissions. Aggression peaked during periods of strictest ward restrictions, while 28-day readmission rate was the strongest overall predictor of incident rate.

**Conclusions:**

By leveraging COVID-19 restrictions as a naturalistic experiment, this study offers a rare temporal and contextualised insight into how systemic factors shape risk in inpatient settings. Findings suggest that loss of autonomy and community leave may increase aggression risk, and repeated admissions may signal escalating self-harm risk. The results underscore the need for further research and development of trauma-informed inpatient care models that support transition to community through flexible care planning and sustained post-discharge support.

**Disclosure of Interest:**

None Declared

